# Supplementary material for: Enhancing Endothelial Differentiation of Mesenchymal Stem Cells Derived from Human Turbinates Using Lab-on-a-Chip Technology
Source: Medicina (Kaunas). 2025 Mar 18;61(3):528. doi: 10.3390/medicina61030528 (PMC11943588; doi:10.3390/medicina61030528)
Supplement: Supplementary file 1 [file medicina-61-00528-s001.zip › medicina-3492163-supplementary.pdf]

medicina

MDPI

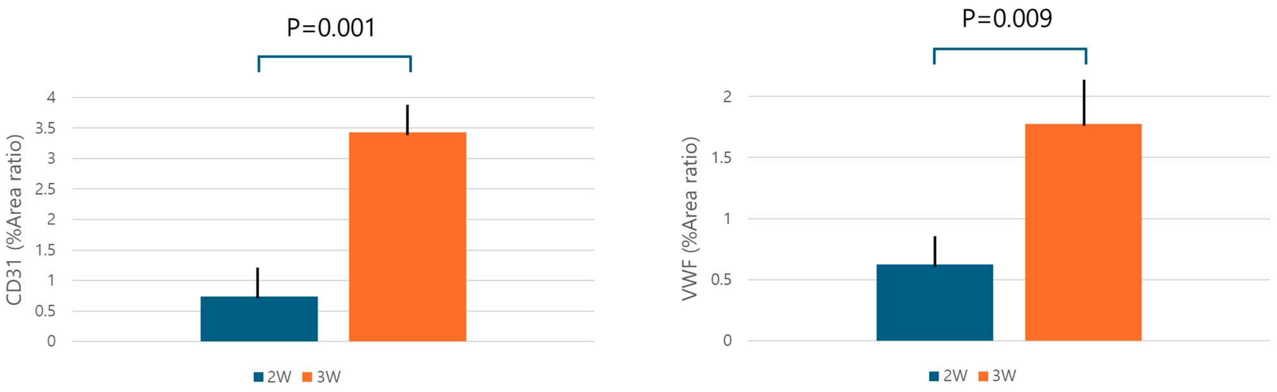

Supplementary Figure S1. Panels graphically represent the percentage area of CD31 and VWF immunostaining after 2 and 3 weeks of culture as determined by ImageJ. Error bars represent standard deviation.

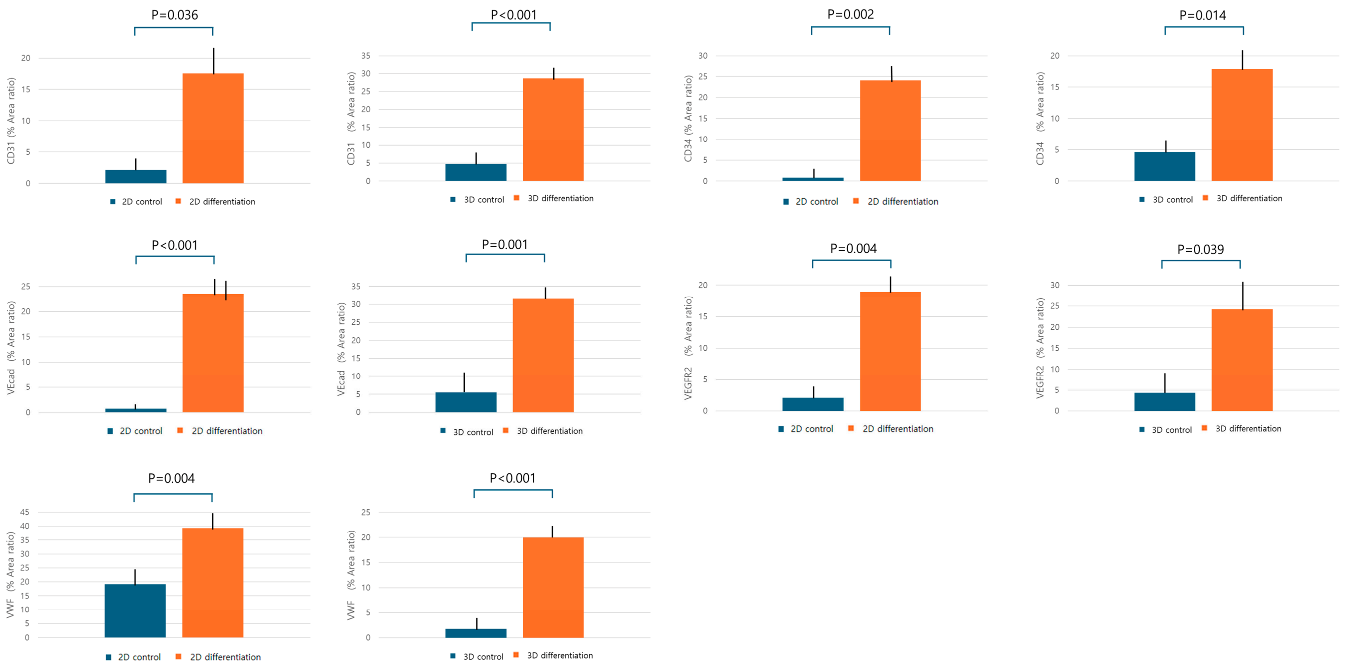

Supplementary Figure S2. Panels graphically show the area ratios (%) of CD31, CD34, VECAD, VEGER2, and VWF immunostaining of 2D and 3D cultures as determined by ImageJ. Error bars are standard deviations.
